# Demography and Risk Factors of Suicide in Bangladesh: A Six-Month Paper Content Analysis

**DOI:** 10.1155/2017/3047025

**Published:** 2017-10-10

**Authors:** Md. Mohsin Ali Shah, Srijony Ahmed, S. M. Yasir Arafat

**Affiliations:** Department of Psychiatry, Bangabandhu Sheikh Mujib Medical University, Dhaka, Bangladesh

## Abstract

**Background:**

Suicide is a global public health problem too often neglected by researchers and Bangladesh is not an exception. There is no suicide surveillance and nationwide study is yet to be conducted in the country.

**Objectives:**

This paper aimed to look into suicide based on newspaper reporting in Bangladesh focusing on the demographic variables and risk factors.

**Methods:**

6 national newspapers were scrutinized from November 2016 to April 2017. Data were checked, cross-checked, and then analyzed with SPSS software.

**Results:**

In a duration of six months, a total of 271 cases were reported; age was found to be in the range of 11–70 years (26.67 ± 13.47). 61% of the reported cases were below 30 years of age, 58% were female, 24% were students, 17% were house makers, 61% were from rural background, and 45% were married. Hanging was found to be the commonest method (82.29%); marital and familial discord remained a noticeable risk factor (34.32%). Family members and neighbors noticed 103 cases, and only 3 cases were found to have suicide notes.

**Conclusion:**

Suicide is an underattended public health problem in Bangladesh with few researches and paucity of literature. Establishment of national suicide surveillance is now a time demanded step.

## 1. Introduction

Suicide is a neglected preventable public health problem across the globe and Bangladesh is not an exception [1]. About one million people die each year by suicide over the world with a global mortality rate of 16 per 100,000 and 39.6 per 100,000 in Bangladesh [1, 2]. Although suicide can happen at any stage of life, it is the second most frequent and in some countries it is the leading cause of death among people aged 15–24 years [1–3]. Developing countries account for about 73% of the global suicides and, on the continents level, Asia accounts for about 60% of global suicides [1, 2]. Bangladesh, being a densely populated developing country in Southeast Asia, has achieved health-related Millennium Developmental Goals (MDG) but suicide is still underaddressed [1, 4]. There is no surveillance for suicide and nationwide study on suicide is yet to be conducted [1, 4]. Moreover, suicide is considered as a criminal offence and religious factors and social factors as well as legal consequences hinder the suicide disclosures [1, 5–8]. The source of information of suicide is mainly based on police report, forensic reports, media reports, and few other institutional reports such as those from hospitals and courts [1, 5]. People prefer to hide suicide news, as it is a criminal offence and aftermath of legal hazards is miserable for the family members. There is also dearth of research and paucity of literatures on suicide of this huge population. Suicide happens because of multifactorial involvement such as genetic, psychological, social, and cultural risk factors; those can interact in different perspectives such as diathesis-stress model and gene-environment interaction [3]. Psychiatric morbidities play an important role in suicide as previous research mentioned that about 90% of suicide victims were found to have at least one psychiatric disorder and depression was the most mentionable one [9, 10]. In Bangladesh, previous studies found variations in risk factors and psychological autopsy is still yet to be conducted. This paper aimed to look into the suicide based on the contents of newspaper reporting in Bangladesh focusing on the demographic variables and risk factors of suicide.

## 2. Methods

### 2.1. Data Collection

6 national daily newspapers were selected purposively to be included in the study and those were scrutinized from November 2016 to April 2017. Among them, 4 were Bangla newspapers (the Daily Jugantor, the Daily Ittefaq, Kaler Kantho, and Daily Prothom Alo) and two were English newspapers (the Daily Star and the Independent). The six papers were collected regularly and initial screening was conducted. Then those were checked by Srijony Ahmed and S. M. Yasir Arafat and approved for collection by cutting the selected parts. From the selected parts, data were organized along with the variables and written in master sheet. Then those were cross-checked by Srijony Ahmed and S. M. Yasir Arafat by comparing the collected parts of the papers and the master sheet. After that, final approval was made by Md. Mohsin Ali Shah and data were considered as mature to give software input. A total of 271 pieces of data were collected and analyzed by Statistical Package for Social Sciences (SPSS) version 16 software and Microsoft Excel version 2007 software. Data were randomly checked by Md. Mohsin Ali Shah at any stage to ensure the correctness.

### 2.2. Inclusion of News

The following news reports were included:

  News that were clearly indicated as suicide
  Suicidal news of only Bangladeshi citizens
  Suicidal news bounded by the geographic area of Bangladesh

### 2.3. Variables

Age, sex, occupation, educational status, residence, religion, nuptial status, method of suicide, identifiable risk factors, time of suicide, noticing/reporting person, suicidal notes, any history of previous attempts, postmortem information, persons involved in suicide, association with psychiatric disorders, reporting variation, and numbers of papers reporting the news were considered as the variables of the study.

### 2.4. Permission

As the data includes only the printed and published information, no formal ethical clearance was needed.

## 3. Results

In a duration of six months, a total of 271 cases were reported; age of the respondents was found to range from 11 to 70 years (26.67 ± 13.47). 61% (83) of the reported cases were below 30 years of age, 58% were female, 11% were under SSC, 24% (64) were students, and 17% were house makers (Table 1). 61% of the cases were from rural background, and 45% were married (Table 1). As the study was based on the content analysis of the newspapers, a significant portion of the variables of a particular case were missing. Among them, age was not found in 20% (53) of the reports, marital status was not found in 19% of the reports, occupation was not found in 32% of the reports, and educational status was not found in 79% of the reports (Table 1). Hanging was found to be the commonest method of suicide: 223 (82.29%) of the respondents committed suicide by hanging (Table 2). Marital and familial discord was found to be the commonest risk factor and 93 (34.32%) of the respondents had discords with spouse or family members before death (Table 2). 82 (32.26%) of the suicides happened at night-time (Table 2). Family members and neighbors noticed 103 (38%) cases; 3 people were found to have suicide notes; 1 report was found to have history of previous suicidal attempt; 1 report was found to have psychiatric illness; 2 reports were found to have history of substance abuse (Table 2). More than one person was involved in 7% (18) of the cases (Table 2). Again, regarding method, 13 (4.8%) reports did not mention it; time was not mentioned in 62 (22.88%) of the reports; risk factors could not be sorted in 130 (48%) reports and informing person was not mentioned in 163 (60%) reports (Table 2). 23 (9%) cases were mentioned to have mental disorders before suicide; 204 (75%) cases were reported in single paper; 41 (15%) had variations in reporting between different papers. Most reports were available in the month of November, 55 (20%); most reports were available in the daily paper “the Daily Jugantor,” 91 (34%), and most reports were found in Bangla newspapers, 228 (84%) (Table 3).

## 4. Discussion

As suicide is still underexplored in Bangladesh, this paper aimed to look into the contents of 6 national newspapers reporting suicide news focusing on the demography and risk factors of suicide in Bangladesh. The study revealed that the age range was 11–70; mean ± SD was 26.67 ± 13.47 years, which signifies the suicides in both marginal ages and mean age signifies the loss of lives in the productive age. Similar finding was reported in the recent review in Bangladesh by Arafat (2017) [1] and Million Death Study by Patel et al. (2012) revealed that the vulnerable age was between 15 and 69 years [11]. However, among the 271 respondents, the age of about 20% of them was missing (Table 1). In 61% of the suicides, age was found to be below 30 years and 24% (64) were students, which supports the vulnerability of age group of 15–24 years and reinforced the productive life losses. In different parts in the world, older age groups are more prone, but in Bangladesh the trend is reversed. Similar findings were found in the recent review in the country [1]. The result revealed that more females (58%) died than males, which is also in contrast to the developed countries' trends but is supported by previous reviews in the same culture [1, 5, 8]. Factors that are considered responsible are passive gender role, low education, lack of economic freedom, early marriage, sociocultural attitude to the females, and male dominance [1]. The study revealed that suicide was more common in married persons (45% married; 35% unmarried; others missing) and was also more common (61%) in rural areas (Table 1) and the result was found to be similar to previous reviews and evidences [1, 5, 8, 11, 12]. In developed countries, males are prone to suicide, but in Bangladesh females are more prone to suicide. Jordans et al. [12] and Patel et al. [11] found that the male-to-female ratio is smaller in Asia than in other parts of the world [11, 12]. In the western parts of the world, marriage is considered as a protective factor, but in Bangladesh repeated articles reported the reverse phenomenon [1, 5–8], which can be explained by socioeconomic structure of this specific area. Hanging was found to be the commonest method of suicide, 223 (82.29%) (Table 2); the finding correlates with other global findings [1, 9, 10]. Previous review revealed larger proportion of suicides caused by poisoning [1, 11]. The finding can be explained by considering the data source of the current study. Being an agriculture-based country, the percentage of suicides by poisoning might be high and as the study considered the contents of print media, the finding might be unexplored. The time interval between death and ingestion of poisons is more than the suicide by hanging, and after noticing the poisoning, people rush to the hospitals. Due to these procedures, paper reporting of suicides by poisoning might be less than the hanging. Moreover, poisoning is more prevalent in rural areas and media focus more on the urban areas. Risk factors were not mentioned in about half of the reports (48%) (Table 2). Among the mentioned risk factors, marital discord (10.33%) and familial disharmony (22.14%) covered the lion's share of the risk factors (Table 2), which is concurrent with reviews in similar culture in which quarrel between spouses was the most common risk factor of suicide [1, 5–8]. Further reported risk factors were sexual harassment, failure in examination, affair break-up, domestic violence, financial constraints, and physical illness (Table 2). Surrounding people (family members and neighbors) noticed the events of suicide as found in the study (Table 2). 23 cases were found to have mental disorders before suicide and that finding is difficult to justify but is similar to previous reviews [1–3, 5–10]. Previous reports revealed that mental health is still poorly considered and addressed in association with suicides. Moreover, people used to hide the mental illness and most of the reporters are not comfortable to excavate the relationship between mental illness and suicide. Majority of the reports (75%) were published in a single newspaper and 15% of the reports varied from newspaper to newspaper with respect to different variables of a particular case such as age, educational status, and occupation (Table 3). News of famous person's suicide is usually covered in multiple newspapers with larger area and English dailies seemed to be reluctant to publish the suicide news.

As only six papers were scrutinized for six months in only printed version, the study's finding may be a snapshot of the movie, but, to the best of the authors' knowledge, this is the first paper content analysis on suicide in Bangladesh. Further larger-scale studies would help to fill up the huge information gap on suicide research in Bangladesh.

## 5. Conclusion

Bangladesh is a populated developing country with high suicide rates. Suicide is still a neglected and underattended public health problem in the country with few researches. Establishment of national suicide surveillance is now a time demanded step to assess the need scientifically as well as to take necessary steps to address it.

## Figures and Tables

**Table 1:** Distribution of demographic variables of the respondents (*n* = 271).

Demographic variable	Frequency	Percentage
Age in years		
11–20	91	33.58
21–30	75	27.68
31–40	20	7.38
41–50	17	6.27
51–60	8	2.95
61–70	7	2.58
Missing	53	19.56
Sex		
Male	113	41.70
Female	158	58.30
Marital status		
Married	123	45.39
Unmarried	95	35.06
Divorced	2	0.74
Missing	51	18.82
Habitat		
Urban	101	37.27
Rural	165	60.89
Occupation		
Doctor	2	0.74
Service holder	13	4.80
Businessman	6	2.21
Laborer	37	13.65
Teacher	2	0.74
Student	64	23.62
House maker	46	16.97
Politician	2	0.74
Others	6	2.21
Law force	4	1.48
Media worker	1	0.37
Missing	88	32.47
Education		
Secondary	31	11.44
SSC	6	2.21
HSC	3	1.11
Graduate	14	5.17
Postgraduate	3	1.11
Missing	214	78.97

**Table 2:** Distribution of suicide variables of the respondents (*n* = 271).

Suicide variable	Frequency	Percentage
Method		
Hanging	223	82.29
Poisoning	21	7.75
Falling from height	2	0.74
Jumping under train	6	2.21
Gun fire	3	1.11
Cutting throat	1	0.37
Drowning	2	0.74
Missing	13	4.80
Time of suicide		
Morning (6 am–12 am)	47	17.34
Evening	30	11.07
Noon (12 pm–4 pm)	30	11.07
Afternoon (4 pm–6 pm)	20	7.38
Night (6 pm–12 am)	65	23.99
Late night (12 am–6 am)	17	6.27
Missing	62	22.88
*Total*	*271*	*100*
Risk factors		
Marital discord	31	11.44
Familial Disharmony	62	22.88
Problem in workplace	1	0.37
Sexual harassment	13	4.80
Financial constraints	6	2.21
Affair	15	5.54
Fail in exam	2	0.74
Domestic violence	6	2.21
Divorce	3	1.11
Physical illness	1	0.37
Missing	131	48.34
Informant		
Law force	1	0.37
Reporter	4	1.48
Neighbors	38	14.02
Family member	65	23.99
Missing	163	60.15

**Table 3:** Distribution of reporting variables of the respondents (*n* = 271).

Reporting variable	Frequency	Percentage
Month		
November	55	20.30
December	33	12.18
January	52	19.19
February	42	15.50
March	47	17.34
April	42	15.50
Name of the paper		
The Daily Jugantor	91	33.58
The Daily Ittefaq	45	16.61
Kaler Kantho	35	12.92
The Independent	17	6.27
Daily Prothom Alo	14	5.17
The Daily Star	9	3.32
Multiple	60	22.14
Language		
Bengali	228	84.13
English	29	10.70
Both	14	5.17
